# 

**Published:** 1909-12-18

**Authors:** 


### Gifts and Bequests.

Mr. Thomas Hughes has left £100 to the North Staffordshire Infirmary.

Mr. George Older Worters has left by will £100 to St. Bartholomew's Hospital.

The will of Mr. Thomas Westbrook Terrey contains a bequest of £100 to the Royal Southampton and South Hants Hospital.

The will of the Rev. Claud Campbell contains bequests of £100 each to the Warneford Hospital, Leamington, and the Midland Home for Incurables, Leamington.

Mrs. Theresa Otterbourg has left £50 to the Home and Hospital for Jewish Incurables, High Road, South Tottenham. She has also left £250 to Dr. Adler, to found and endow a bed in the New Jewish Hospital in Jerusalem. The residue of her estate, subject to specific bequests, she has left to the David Lewis Northern Hospital in Liverpool, to found a ward, or, should this be impracticable, to found a home in Liverpool for women.

Mr. John Barton, of Basingstoke, has left £50 to the Basingstoke Cottage Hospital.

Mr. John Toner, if the residue of his property after certain bequests shall exceed £7,100, has left £200 (or the amount of such excess) to the Brompton Hospital for Consumptives.

A cheque for £200, as a further sum from the executors of the late Miss M. J. Magee on account of her residuary bequest has been received by the Governors of the Rotunda Hospital, Dublin.

The Corporation of the City of London has made the following grants: To the Great Northern Central Hospital, the Cancer Hospital, and Seamen's Hospital, Greenwich, £100 each; London Lock Hospital, £75; University College Hospital, Queen Charlotte's Hospital, National Hospital for the Paralysed and Epileptic, Royal Sea-Bathing Hospital, Margate, £50 each; Lowestoft Hospital, Belgrave Hospital, £25 each.

### Appointments.

Dr. J. B. Richardson has been appointed as medical officer to the Shawbury district.

Dr. Byham has been appointed medical officer of health to the Sunbury Urban District Council.

Dr. J. O'Connor has been appointed medical officer of health to the Lutterworth Rural District Council.

Mr. Cecil Rowntree, M.B., B.S., F.R.C.S., has been appointed assistant surgeon to the Cancer Hospital.

Dr. F. T. Bond has been reappointed medical officer of health to the Chipping Sodbury Rural District Council.

Dr. Oswald F. Rowley, of Victoria Road, Barnsley, has been appointed medical officer to the Penistone Board of Guardians.

Dr. Clements, medical officer of health and medical inspector of schools for Batley, has been appointed medical officer of health for Beckenham.

### Appointments Vacant.

Edmonton Union.—Resident medical superintendent.

Royal Albert Hospital, Devonport.—Resident medical officer.

Manchester Children's Hospital.—Assistant medical officer.

### THE QUESTION BOX.

SPECIAL NOTICE.—Questions of interest affecting the honorary medical staff and hospital administration in all departments are invited. When any point arises we hope our readers will formulate it in a question and so co-operate to make this column of real value and interest. In this way the experience of the best-managed hospitals should be made helpful to the whole hospital world. We shall welcome the contribution of both questions and answers.

N.B.—The publication of an answer in this column does not necessarily preclude the publication of subsequent answers to the same question, for a question may involve points which require to be threshed out and fully discussed. Answers, if published, will be paid for at scale rates.

### LADIES' GUILDS AND COMMITTEES.

#### HOW TO HELP A GENERAL HOSPITAL.

QUESTION 1: How can ladies best help a general hospital?

**A Cardiff View.**

By Mr. LEONARD D. REA, Secretary and General Superintendent, Cardiff Infirmary.

There is scarcely a more effective way by which ladies can help a general hospital than by the formation of a Needlework or Linen Guild.

**A Coventry View.**

By Mr. J. A. RUDD, Secretary, Coventry and Warwickshire Hospital.

A ladies' committee has been run in connection with this hospital for the past eight years, and the work done has largely augmented the annual income of the hospital.

#### HOW TO FORM A NEEDLEWORK GUILD.

QUESTION 2: What are the best lines on which to run ladies' guilds and committees?

**A Cardiff View.**

By Mr. LEONARD D. REA, Secretary and General Superintendent, Cardiff Infirmary.

The object of a Needlework Guild is to supply clothing for the use of patients whilst in hospital. It is important to secure the services of a popular and influential lady as President, who should be assisted by a committee of about twenty ladies, who may, if considered desirable, represent the various districts which the hospital serves. Each committee lady has her own associates, as many as she likes. Practically the only rule is that an associate is to contribute not less than two of the required garments each year. The garments are jackets, nightgowns, stockings, etc., as described on lists supplied to the associates. A Linen Guild may be formed on similar lines, either independently or in association with the Needlework Guild. The object of a Linen Guild is to supply linen for the use of the hospital, each associate contributing not less than two articles. Care has to be taken that uniformity in size and quality of articles is insisted upon. This is secured by a carefully compiled list of the articles required—sheets, draw-sheets, etc.—giving sizes and other particulars, and an arrangement may be made with local tradesmen to keep in stock the articles required.

**A Coventry View.**

By Mr. J. A. RUDD, Secretary, Coventry and Warwickshire Hospital.

To run a ladies' committee successfully it is most essential that an energetic secretary be appointed. In this respect the ladies' committee in connection with this hospital is very fortunate. The city and neighbourhood are divided into districts, and a district is taken by each lady and an annual collection made. Last year the amount received from this source was over £279, which is a record collection, and £31 more than the amount collected the previous year. This committee is represented on the General Committee by three members elected annually, one of whom is always the secretary. Last year a Sewing Guild was formed in connection with this committee, and they handed over to the matron 139 articles of clothing, which have greatly added to the comfort of the patients. This committee is invaluable to the work of the hospital, and the inaugurating of such a committee is well worth the while of the committees of management of other institutions.

#### LADIES' GUILD MEETINGS.

QUESTION 3: Is it feasible for ladies to meet at the hospital once a fortnight and do needlework on the premises?

**A Cardiff View.**

By Mr. LEONARD D. REA, Secretary and General Superintendent, Cardiff Infirmary.

ANSWER: No.

I do not think it is feasible or expedient for ladies to meet at the hospital periodically and do needlework on the premises; but the Guilds should meet at the hospital at least once a year, when a report should be submitted as to the number of articles supplied, etc. If held in the summer, such meeting may take the form of a garden-party, the expenses of which can easily be defrayed by a small subscription (say 5s.) from each committee lady. This makes a pleasant opportunity for all associates to meet together, when, in addition to the reports as to the working of the Guilds, one or two short effective speeches should be made, describing the work of the hospital generally, whilst the wards and various departments should also be visited.

I venture to predict, from my experience, that by such simple arrangements, adapted to meet local requirements or the particular circumstances of each institution, a hospital will not only be adequately supplied from year to year with clothing for its patients and linen for its wards, but a widening interest will also be awakened in its general work, the expression of which will not be limited by the provision of linen or clothing.

### THE METROPOLITAN HOSPITALS.

Dr. E. ROWLAND FOTHERGILL, M.B., B.S., submits the following queries which we publish with the answers in each case:

1. What steps are at present taken by the large hospitals of London to prevent imposition?

[The appointment of special officers of inquiry is now pretty general. These officers are required to devote their whole time to the checking of abuse and inquiry into the circumstances of the patients.]

2. If it is at all by almoners, how many of these are attached to each hospital?

[Some hospitals employ one and some two almoners, or, as we prefer to call them, inquiry officers. It depends upon the volume of work to be overtaken.]

3. How do they ensure that replies to queries are correct?

[By inspection of the patients' homes and oral and written inquiries about the patients.]

4. What percentage of the attendances at out-patients' departments are investigated by them?

[It is not a question of percentages, but of patients. The best system deals with every patient applying for treatment.]

5. How are these almoners able to determine that a would-be patient is "suitable for hospital treatment," *from the aspect of his disease?* as I presume they are not medical men or women.

[This point does not arise in the course of an inquiry officer's duties. He reports on each case on its merits, and where there is any such question as that mentioned the physician or surgeon in attendance determines the point.]

